# Aquaculture and Florfenicol Resistance in *Salmonella enterica* Serovar Typhimurium DT104

**DOI:** 10.3201/eid1504.081171

**Published:** 2009-04

**Authors:** Felipe C. Cabello

**Affiliations:** New York Medical College, Valhalla, New York, USA

**Keywords:** Aquaculture, florfenicol resistance, Salmonella enterica serovar Typhimurium, letter

## To the Editor

In a letter recently published in Emerging Infectious Diseases, Smith (*1*) discussed evidence that he mistakenly believes to undermine the hypothesis that the florfenicol resistance gene present in some isolates of the epidemic *Salmonella enterica* serovar Typhimurium DT104 strain originated from a florfenicol resistance plasmid present in *Vibrio damsela* (*Pasteurella piscicida*) that infected fish farms in Japan in the 1990s (*2*). Smith correctly states that the florfenicol resistance gene was present in *S*. *enterica* serovar Typhimurium DT104 strains isolated in the United States in 1985, before the gene was documented in *V*. *damsela* in Japan (*1**,**3*). He is also correct in noting that this particular florfenicol resistance gene was detected in a plasmid in *Klebsiella pneumoniae* in France in 1969 (*1**,**4*).

However, an earlier report by Briggs and Fratamico (*5*) clearly established that the florfenicol resistance genes and the tetracycline resistance genes *tetG* and *tetR* in the *Salmonella* genomic island 1 (SGI1) were surrounded by non–antimicrobial-drug resistance DNA. This DNA is homologous to DNA sequences in plasmids PASPPFLO and pJA8122 (see Figure 1 and Table 2 in reference *5*) (*5**–**7*). In addition to antimicrobial drug resistance genes, PASPPFLO and pJA8122 contain cloned DNA segments of indigenous R plasmids found in *V*. *damsela* and *V*. *anguillarum*, respectively; these cloned DNA segments span sequences that extend beyond their florfenicol resistance and *tetR*/*tetG* genes (*5**–**7*). For example, the region of the florfenicol resistance gene in SGI1 contains 763 nt of the non–antimicrobial-drug resistance portion of the original *V*. *damsela* plasmid; the region of *tetR*/*tetG* contains 468 nt of the non–antimicrobial-drug resistance DNA segment of the *P*. *piscicida* plasmid (*5**–**7*).

The presence of these non–antimicrobial-drug resistance R plasmid DNA sequences in SGI1 constitutes a molecular signature that firmly establishes the aquaculture origin of the florfenicol resistance and the *tetR*/*tetG* genes in the *S*. *enterica* serovar Typhimurium DT104 strain studied by Briggs and Fratamico and in the SGI1 of other bacteria (*5*). These R plasmid DNA sequences in SGI1 also confirm direct or indirect horizontal gene transfer between bacteria in the aquaculture environment and *S*. *enterica* serovar Typhimurium DT104 (*5**–**7*).
