# Supplementary material for: Aquaculture and Florfenicol Resistance in Salmonella enterica Serovar Typhimurium DT104
Source: Emerg Infect Dis. 2009 Apr;15(4):623–4. doi: 10.3201/eid1504.081171 (PMC2671426; doi:10.3201/eid1504.081171)
Supplement: Related texts

# Reply: In Response

**Authors:** Peter Smith

**Affiliations:** National University of Ireland, Galway, Ireland

In his letter (*1*), Cabello makes 2 observations regarding the debate concerning the origin of the floR gene in Salmonella enterica serovar Typhimurium DT104. The first observation is that the plasmid PASPPFLO contained cloned segments of an indigenous Vibrio damsela plasmid. However, PASPPFLO is not the name of a plasmid but is the GenBank locus identifier associated with the sequence (GenBank accession no. D37826) of a 3,745-bp region of the V. damsela plasmid pSP92088 that contained pp-flo (*2**,**3*).

The second observation is that sequences flanking the *floR* gene in *S*. *enterica* serovar Typhimurium DT104 (GenBank accession no. AF071555) are homologous to those flanking the pp-*flo* gene sequenced from the *V*. *damsela* plasmid pSP92088 (*4*). On the basis of this homology, he seems to assume that these flanking sequences must have originated in *V*. *damsela* and, therefore, that they constitute a molecular signature that firmly establishes the aquaculture origin of this florfenicol resistance. What Cabello does not mention is that sequences flanking a wide range of *floR* genes, including those in plasmid R55 (GenBank accession no. AF332662), are also homologous to those found in *S*. *enterica* serovar Typhimurium DT104 (*5*,*6*).

These data suggest that during horizontal transfer between species and genera, the association of *floR* with its flanking regions has been conserved (*5*,*6*). However, the data provide no evidence for postulating a unique association of these flanking sequences with *V*. *damsela*, and, therefore, do not provide evidence for an aquaculture origin of *floR*. If Cabello believes that sequences flanking *floR* in *S*. *enterica* serovar Typhimurium DT04 constitute a molecular signature that firmly establishes the aquaculture origin of *floR* in *S*. *enterica* serovar Typhimurium DT104, he should provide some explanation as to how this signature was also present in the R55 plasmid detected in a *Klebsiella pneumoniae* strain isolated in 1969 (*5*,*7*).

## References

[1] Cabello FC. Aquaculture and florfenicol resistance in *Salmonella enterica* serovar Typhimurium DT104. Emerg Infect Dis. 2009;15:623. 10.3201/eid1504.08117119331756PMC2671426

[2] Kim E, Aoki T. Sequence analysis of the florfenicol resistance gene encoded in the transferable R-plasmid of a fish pathogen *Pasteurella piscicida.* Microbiol Immunol. 1996;40:665–9.890861210.1111/j.1348-0421.1996.tb01125.x

[3] Kim EH, Yoshida T, Aoki T. Detection of R plasmid encoded with resistance to florfenicol in *Pasteurella piscicida.* Fish Pathology. 1993;28:165–70.

[4] Briggs CE, Fratamico PM. Molecular characterization of an antibiotic resistance gene cluster of *Salmonella typhimurium* DT104. Antimicrob Agents Chemother. 1999;43:846–9.1010318910.1128/aac.43.4.846PMC89215

[5] Cloeckaert A, Baucheron S, Chaslus-Dancla E. Nonenzymatic chloramphenicol resistance mediated by IncC plasmid R55 is encoded by a *floR* gene variant. Antimicrob Agents Chemother. 2001;45:2381–2. 10.1128/AAC.45.8.2381-2382.200111451703PMC90660

[6] Schwarz S, Kehrenberg C, Doublet B, Cloeckaert A. Molecular basis of bacterial resistance to chloramphenicol and florfenicol. FEMS Microbiol Rev. 2004;28:519–42. 10.1016/j.femsre.2004.04.00115539072

[7] Chabbert YA, Scavizzi MR, Witchitz JL, Gerbaud GR, Bouchaud DH. Incompatibility groups and the classification of fi-resistance factors. J Bacteriol. 1972;112:666–75.462874410.1128/jb.112.2.666-675.1972PMC251473
